# Performance of Silicone Rubber Composites Filled with Aluminum Nitride and Alumina Tri-Hydrate

**DOI:** 10.3390/ma13112489

**Published:** 2020-05-29

**Authors:** Jianjun Zheng, Shaojian He, Jiaqi Wang, Wenxuan Fang, Yang Xue, Liming Xie, Jun Lin

**Affiliations:** 1 Inner Mongolia Electric Power Science & Research Institute, Hohhot 010020, China; dkyzjj@163.com (J.Z.); dlfwx1994@163.com (W.F.); nmdkyxlm@163.com (L.X.); 2 School of Renewable Energy, North China Electric Power University, Beijing 102206, China; heshaojian@ncepu.edu.cn (S.H.); jqw9542@163.com (J.W.); 3 State Key Laboratory of Multi-Phase Complex Systems, Institute of Process Engineering, Chinese Academy of Sciences, Beijing 100190, China

**Keywords:** silicone rubber, aluminum nitride, alumina tri-hydrate, vinyl tri-methoxysilane, dielectric loss, breakdown strength

## Abstract

In this study, silicone rubber (SR) composites were prepared with various amounts of aluminum nitride (AlN) and alumina tri-hydrate (ATH), and vinyl tri-methoxysilane (VTMS) was also introduced to prepare SR/ATH/AlN–VTMS composites for comparison. Compared to the SR/ATH composites, the SR/ATH/AlN composites with higher AlN loading exhibited higher breakdown strength and thermal conductivity, which were further improved by the addition of VTMS. Such results were related to the enhanced rubber–filler interfacial interactions from VTMS coupling, as demonstrated by scanning electron microscopy (SEM) analysis and the curing behaviors of the SR composites. Moreover, by replacing ATH with VTMS-coupled AlN, the SR/ATH/AlN–VTMS composites also exhibited lower dielectric loss along with an increased dielectric constant, suggesting the promising application of VTMS-coupled AlN as a filler for the preparation of the SR composites as high-voltage insulators.

## 1. Introduction

High-temperature-vulcanized silicone rubber (SR) is widely used to manufacture high voltage insulators due to its relatively light weight and excellent anti-contamination ability [1,2,3,4]. It is often filled with suitable additives so that the performance of the SR composites can be improved [5,6,7,8,9,10,11]. To date, alumina tri-hydrate (ATH) has been one of the most commonly used fillers in SR composites, which can endow the composites with good flame retardancy and erosion resistance. However, under arc discharging, the evaporation of the crystal water from ATH at over 220 °C can increase the roughness of the composite surface, which would be more inclined to be wet-out, and result in dry band arcing [12,13,14]. Therefore, alternative filler materials including boron nitride (BN), mica, aluminum oxide, etc. have been sought to replace ATH in high voltage SR composite insulators [15,16,17,18,19].

By incorporating 7 wt.% thermally conductive BN particles in the SR composites, Du et al. [20] found an improvement in the thermal dissipation and a decrease in both erosion depth and weight loss, indicating improved electrical erosion resistance. By the addition of mica modified by polydopamine and silane coupling agents, Liao et al. [16] reported SR composites with ~43% higher breakdown strength as compared to that of the ATH-filled SR composites. Aluminum nitride (AlN) is a thermally conductive material with a combination of good electrical insulation ability and low dielectric loss, which is desired for applications in high voltage insulation. However, to the best of our knowledge, the use of AlN in high-voltage SR composites has rarely been reported, even though AlN has been used to prepare various polymer composites [21,22,23,24,25]. Therefore, it would be of great importance to find out whether AlN would be a promising filler to replace ATH for the preparation of high-voltage insulating SR composites.

In this work, SR composites with various amount of AlN and ATH were prepared. Since the filler–rubber interfacial compatibility significantly influences the performance of the composites, a commonly used silane coupling agent, vinyl tri-methoxysilane (VTMS), was also introduced, with the intention to strengthen the rubber–filler interfacial interactions. To understand the structure–property relationship in these SR composites, the microstructure, thermal properties, dielectric properties and mechanical performance were investigated.

## 2. Experimental

### 2.1. Materials

SR (MVQ110, vinyl weight percentage: 0.23%, molecular weight: 680,000 g/mol) was provided by Wynca Chemical Group Co., Ltd. (Xin’an, China). AlN (ZH-AlN010, ~10 μm, 99.0%) was purchased from Hefei Zhonghang Nanometer Technology Development Co., Ltd. (Hefei, China). ATH (2~5 μm, 97.0%) was bought from Zhongke Flame-retardant New Material Co., Ltd. (Hefei, China). VTMS was purchased from Chenguang Chemical Industry Co., Ltd. (Qufu, China). 2,5-bis(tert-butylperoxy)-2,5-dimethyl hexane (DBPMH) was purchased from Meixing Chemical Industry Co., Ltd. (Laiwu, China). All the above-mentioned materials were used as received.

### 2.2. Sample Preparation

Preparation of SR composites: Filler (including ATH and AlN), curing agent and silane coupling agent were mixed with pure SR using a 6 inch two-roll mill to prepare SR compounds, which were then placed in a standard mold and vulcanized using a XLB-D 350 hydraulic hot press (Dongfang Machinery, Co., Ltd., Huzhou, China) at 170 °C and 15 MPa for 10 min to obtain SR composites. The recipe for the SR composite preparation was as follows (parts by weight): SR, 100; filler, 150; DBPMH, 0.5; VTMS, 0 or 1.0. The as-prepared SR composites were named SR/ATHx/AlNy and SR/ATHx/AlNy–VTMS, respectively, where x and y refer to the amounts of the parts of ATH and AlN in the composites, respectively.

### 2.3. Sample Characterization

The curing behaviors of the SR composites were recorded by moving a die rheometer (MDR, D&G Measure Instrument Co. Ltd., Shanghai, China) at 170 °C by following ASTM D5289-2017. A TG-Q500 analyzer (TA Instruments, New Castle, DE, USA) was used to perform thermogravimetric analysis (TGA) with a heating rate of 20 °C min^−1^ under a nitrogen atmosphere. A SU8010 scanning electron microscopy (SEM) (Hitachi Co. Ltd., Tokyo, Japan) was utilized to observe the morphologies of the composites after the tensile tests at an accelerating voltage of 5 kV. A 6500B impedance analyzer (Wayne Kerr Electronics, Shenzhen, China) was used to detect the dielectric constant and dielectric loss. An HCDJC-50kV dielectric strength tester (Beijing Huace Testing Instrument Co. Ltd., Beijing, China) was used to measure the breakdown strength with a stepping voltage of 1 kV/s. To obtain the thermal conductivity (*κ*) of the composites, the specific heat (*C_p_*), density (*ρ*) and thermal diffusivity (*α*) were evaluated by differential scanning calorimetry (DSC, NETZSCH, Selb, Germany) (with a heating rate of 10 °C·min^−1^ under a nitrogen atmosphere), a density tester (MH-300A, MatsuHaku, Xiamen, China) and laser flash apparatus (LFA 427, NETZSCH, Selb, Germany), respectively, and then calculated by the equation *κ* = *α* × *C_p_* × *ρ*. An AI-7000S1 electrical tensile tester (Goodtechwill Testing Machines, Co. Ltd., Qingdao, China) was used to record the stress–strain behaviors with a tensile speed of 500 mm·min^−1^ following ASTM D412-2016. All measurements were performed at 25 °C unless mentioned otherwise.

## 3. Results and Discussion

### 3.1. Curing Behavior

**Figure 1:**
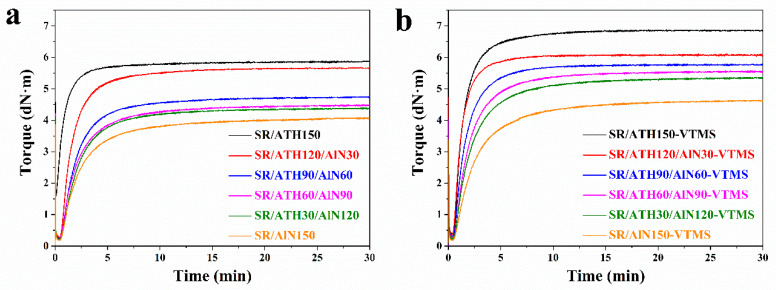
Curing curves of the (**a**) silicone rubber (SR)/alumina tri-hydrate (ATH)/AlN and (**b**) SR/ATH/AlN–vinyl tri-methoxysilane (VTMS) composites.

Figure 1 shows the curing curves of the SR composites with various ATH and AlN contents, in which the torque reaches a plateau at a maximum value during the final stage of the curing process. In general, the torque is determined by the interactions in the rubber nanocomposites, including the rubber–rubber, filler–rubber and filler–filler interactions [26]. For the SR/ATH/AlN composites (Figure 1a), the maximum torque decreases with an increasing AlN content. Such a decrease suggests that the replacement of ATH with AlN could weaken the interactions in the SR composites. On the one hand, the same amount of curing agent was added in these composites, which should result in similar crosslinking density, and thus the rubber–rubber interactions should not change much upon the addition of AlN. On the other hand, as compared to the ATH particles, the AlN particles are larger and have smaller surface areas, which should cause fewer filler–rubber and filler–filler interactions when added in the SR composites. As a result, a lower torque value is expected for the SR/ATH/AlN composites with higher AlN content. In comparison, as shown in Figure 1b, the SR/ATH/AlN–VTMS composites exhibit a higher torque value than the corresponding SR/ATH/AlN composites with the same AlN content, suggesting that the incorporation of the silane coupling agent VTMS strengthens the interactions in the rubber composites.

**Figure 2:**
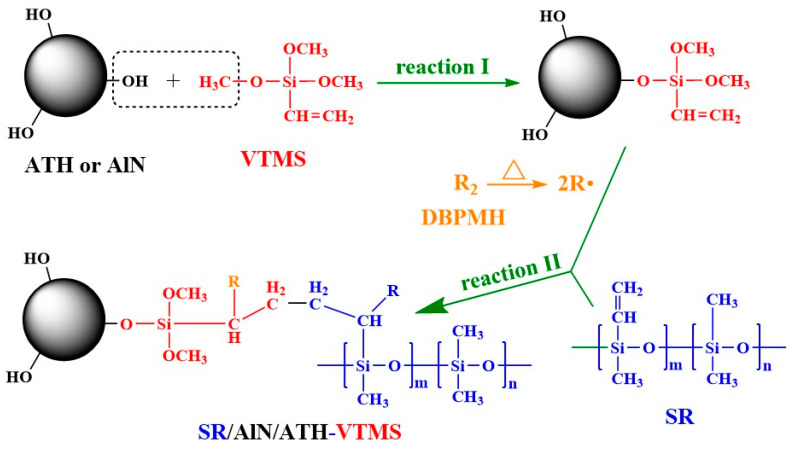
Formation of the SR/BN/ATH–VTMS composite.

During the sample preparation, VTMS was grafted onto the filler particles (ATH and AlN) via the condensation reaction between the hydroxyl groups on the fillers and the alkoxyl groups on VTMS (Figure 2, Reaction I). During the vulcanization process, DBPMH decomposed and produced free radicals, initiating the radical addition reactions between the vinyl groups on both SR and VTMS (Figure 2, Reaction II). Therefore, the VTMS-grafted filler particles were covalently connected to the SR macromolecular chains, leading to the stronger filler–rubber interactions and higher crosslinking density of the rubber network.

### 3.2. TGA Analysis

**Figure 3:**
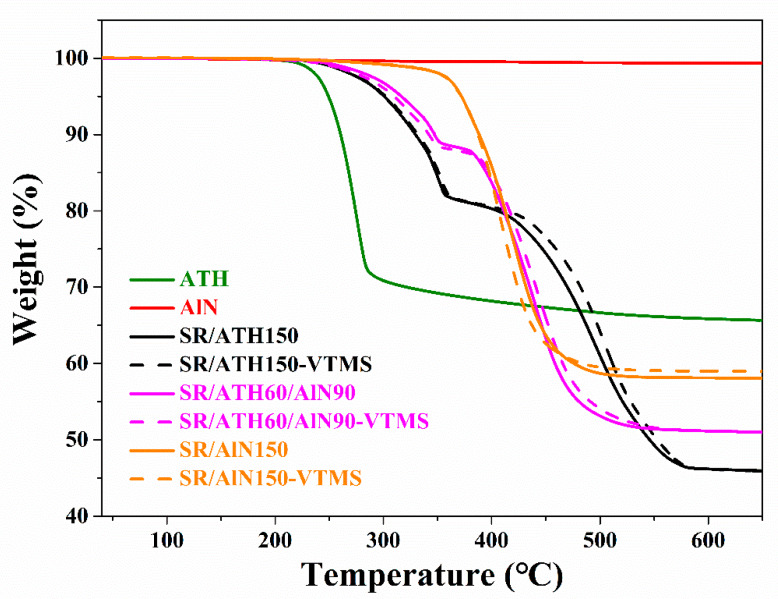
Thermogravimetric analysis (TGA) curves of the AlN, ATH and SR composites.

The TGA curves of the ATH, AlN and SR composites are shown in Figure 3. For the ATH powder, a weight loss of ~34 wt.% starting from ~220 °C can be observed, which is due to the dehydration of the ATH [27]. As for the AlN powder, no weight loss is found in the TGA curve below 650 °C, indicating the excellent thermal stability of AlN. For the SR/ATH150 composite, two steps of weight loss are observed: the first one starting from ~220 °C with ~18 wt.% weight loss is mainly ascribed to the dehydration of ATH, and the second one starting from ~360 °C with ~36 wt.% weight loss results from the decomposition of the SR matrix. The SR/ATH60/AlN90 composite also exhibits a two-stage thermal degradation behavior, with a smaller weight loss between 220 and 360 °C due to the lower ATH loading in the composite than that in the SR/ATH150 composite. By comparison, for the SR/AlN150 composite, there is only one weight loss of ~40 wt.% between 360 and 500 °C, which results from the degradation of the SR, since the weight percentage of SR in the composite is right at 40 wt.%. Therefore, the thermal stability of the SR composites could be enhanced through the filler replacement of ATH by AlN. It is also found that the TGA curves of the SR/ATH/AlN–VTMS composites are very close to those of the corresponding SR/ATH/AlN composites, suggesting that the addition of VTMS does not change the stability of the SR composites.

### 3.3. SEM Observation

**Figure 4:**
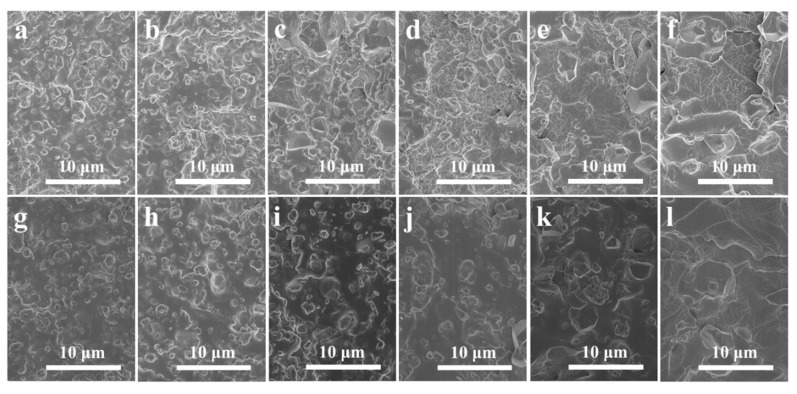
SEM images of the (**a**) SR/ATH150, (**b**) SR/ATH120/AlN30, (**c**) SR/ATH90/AlN60, (**d**) SR/ATH60/AlN90, (**e**) SR/ATH30/AlN120, (**f**) SR/AlN150, (**g**) SR/ATH150–VTMS, (**h**) SR/ATH120/AlN30–VTMS, (**i**) SR/ATH90/AlN60–VTMS, (**j**) SR/ATH60/AlN90–VTMS, (**k**) SR/ATH30/AlN120–VTMS and (**l**) SR/AlN150–VTMS composites.

The tensile fracture surface morphologies of the SR composites with various ATH and AlN contents were observed by SEM. Both ATH and AlN are spherical particles, so the difference between them is the size of the particles. For the SR/ATH/AlN composites shown in Figure 4a–f, there exist some filler particle agglomerations exposed on the tensile fracture surfaces. Due to the larger particle size of AlN than that of ATH, the size of the agglomerations increases with the AlN content. In addition, some voids are also found in the composites, demonstrating relatively weak interfacial adhesion between the fillers and rubber matrix. Meanwhile, for the SR/ATH/AlN–VTMS composites shown in Figure 4g–l, there exist fewer agglomerations and voids, demonstrating that the incorporation of VTMS reduces the filler agglomerations and improves the interfacial interactions between the filler particles and SR matrix.

### 3.4. Dielectric Properties

**Figure 5:**
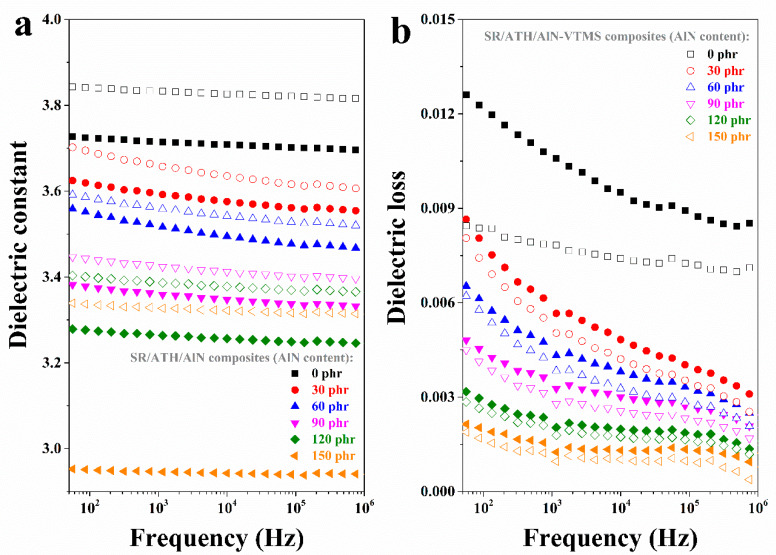
(**a**) Dielectric constant and (**b**) dielectric loss of SR composites.

The dielectric properties of the SR composites with various AlN contents as a function of frequency are shown in Figure 5. Since the polarity of AlN is weaker than that of ATH, the replacement of ATH by AlN could reduce the interfacial polarization for the SR composite. As a result, both the dielectric constant and dielectric loss of the SR composites decrease with an increasing AlN content. As for the SR/ATH/AlN–VTMS composites, it is found that they exhibit a higher dielectric constant and lower dielectric loss than the corresponding SR/ATH/AlN composites containing the same amount of ATH/AlN. VTMS in situ-modified ATH and AlN are more compatible with the SR matrix, so both the interfacial polarization and space charge accumulation in the SR/ATH/AlN–VTMS composites should be reduced as compared to those in the SR/ATH/AlN composites. As a result, a lower dielectric loss is observed for the SR/ATH/AlN–VTMS composites. Meanwhile, the increase in the dielectric constant of the SR/ATH/AlN–VTMS composites could rationalized as follows: the introduction of VTMS not only strengthens the filler–rubber interactions but also raises the composite crosslinking density, which results in lower free volume. Such a decrease in the free volume of the SR composites should lead to an increase in the dielectric constant, which has also been reported previously [28,29,30].

### 3.5. Breakdown Strength

**Figure 6:**
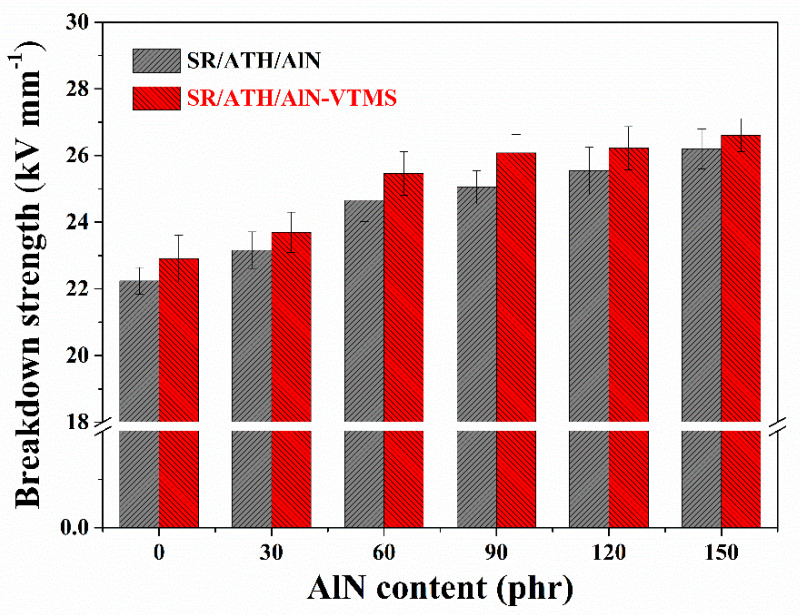
Breakdown strength of SR composites.

The breakdown strength of the SR/ATH/AlN composites with various ATH and AlN contents is shown in Figure 6. It is found that the breakdown strength increases with an increasing AlN content for either the SR/ATH/AlN or SR/ATH/AlN–VTMS composites. The breakdown strength of the SR/AlN150 composite reaches 26.2 kV mm^−1^, ~18% higher than that of the SR/ATH150 composite (22.2 kV mm^−1^). This should be due to the better insulating property of AlN and the lower interfacial polarization for the AlN-filled composites. With the introduction of VTMS, all the SR/ATH/AlN–VTMS composites show higher breakdown strength than the corresponding SR/ATH/AlN composites containing the same amount of AlN. Such a result could be related to the fact that there are fewer structural defects in the SR/ATH/AlN–VTMS composites because of the strengthened rubber–filler interactions from VTMS incorporation, as demonstrated in the above-mentioned SEM results.

### 3.6. Thermal Conductivity

**Figure 7:**
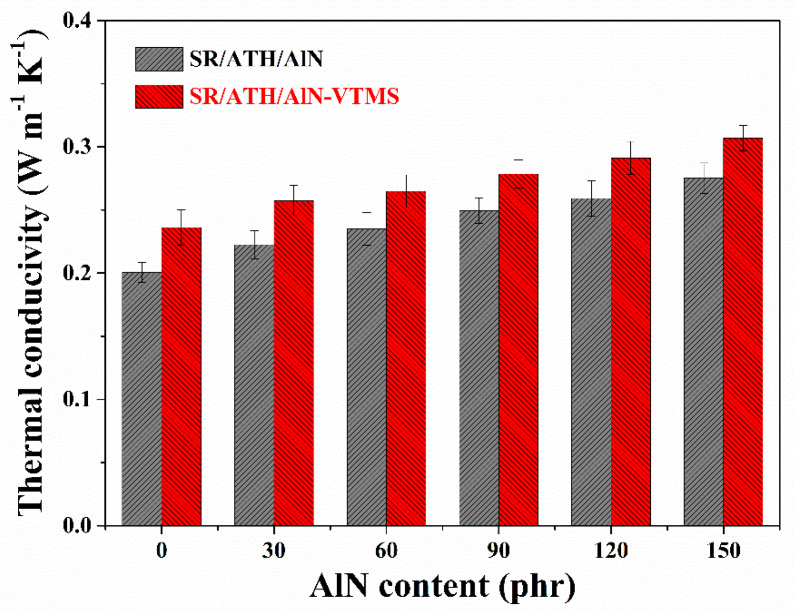
Thermal conductivity of SR composites.

The thermal conductivity of both the SR/ATH/AlN and SR/ATH/AlN–VTMS composites gradually increases with an increasing AlN content (Figure 7), mainly because AlN has higher intrinsic thermal conductivity (~150 W m^−1^ K^−1^) than ATH (~30 W m^−1^ K^−1^). After all of the ATH particles are replaced by AlN, the SR/AlN150 and the SR/AlN150–VTMS composites exhibit thermal conductivities of 0.275 W m^−1^ K^−1^ and 0.307 W m^−1^ K^−1^, respectively, 37.5% and 30.1% higher than those for the SR/ATH150 (0.200 W m^−1^ K^−1^) and SR/ATH150–VTMS (0.236 W m^−1^ K^−1^) composites, respectively. As expected, the introduction of VTMS further enhances the thermal transport properties of the composites, so the SR/ATH/AlN–VTMS composites always exhibit higher thermal conductivity than the corresponding SR/ATH/AlN composites with the same AlN content. As illustrated previously, the addition of VTMS facilitated the strengthening of the filler–rubber interfacial interactions in the SR/ATH/AlN–VTMS composites as compared to those in the SR/ATH/AlN composites, resulting in lower interfacial heat resistance [31]. As a result, in addition to the improved insulation properties, the incorporation of AlN particles along with VTMS into the SR composites could also significantly enhance the heat transfer to effectively dissipate the heat generated within the composites.

### 3.7. Mechanical Properties

**Figure 8:**
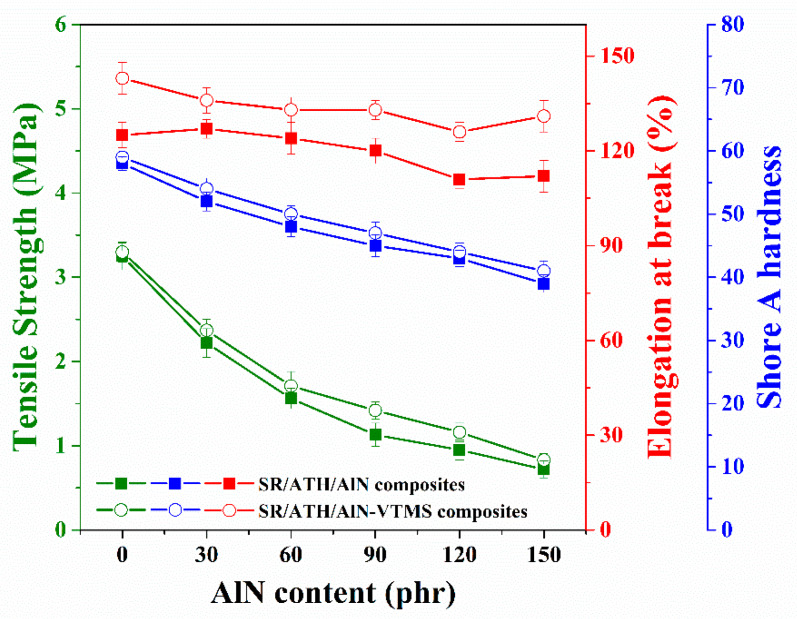
Tensile strength, elongation at break and Shore A hardness of the SR composites.

The tensile strength, elongation at break and Shore A hardness of the SR composites as a function of the AlN content are summarized in Figure 8. As demonstrated from the curing behaviors of these SR composites, the filler–filler and filler–rubber interactions decrease with an increasing AlN content, leading to smaller values of Shore A hardness when the loading of AlN is higher. Besides, due to the larger particle size and smaller surface area of AlN, the rubber chains are less restrained by AlN than ATH. Therefore, the composites with higher AlN content exhibit lower tensile strength and elongation at break. After the incorporation of VTMS, the SR/ATH/AlN–VTMS composites are found to exhibit higher values for tensile strength, elongation at break and Shore A hardness as compared to the SR/ATH/AlN composites with the same AlN content, which is mainly ascribed to the increase in both rubber–filler interfacial interactions and composite crosslinking density facilitated by VTMS. According to JB/T 10945-2010, the SR materials for composite insulators should achieve a tensile strength of 3 MPa and an elongation at break of 100%. Therefore, the tensile strength of the AlN-filled SR composites needs to be improved further to satisfy applications in the high-voltage insulation field.

## 4. Conclusions

In this work, SR composites with various AlN and ATH content were used to prepare SR/ATH/AlN composites, and VTMS was also introduced to prepare SR/ATH/AlN–VTMS composites for comparison. Upon replacing ATH with AlN as the filler, the SR composites demonstrated improvements in thermal stability, breakdown strength and thermal conductivity, while the mechanical properties deteriorated. In addition, higher AlN loading resulted in a reduced dielectric constant and dielectric loss for the SR composites. With the introduction of VTMS, the SR composites exhibited an enhancement of rubber–filler interfacial interactions, as demonstrated by the curing behaviors and SEM morphology analysis. This led to an improvement of the performance of the SR composites, including higher tensile strength and Shore A hardness. Furthermore, the dielectric constants of the SR composites were increased along with a reduction in the dielectric loss after VTMS was added, which could be related to the lower free volume and lower space-charge accumulation at the filler–matrix interface. The breakdown strength and thermal conductivity of SR composites were also further improved by the addition of VTMS. Therefore, our work has demonstrated that AlN coupled with VTMS could be very promising for the preparation of the SR composites to be applied in the field of high voltage insulation as long as we overcome the issue of the decrease in mechanical performance.

## References

[1] Liu P.Y., Li L.C., Wang L.M., Huang T., Yao Y.B., Xu W.R. (2019). Effects of 2D boron nitride (BN) nanoplates filler on the thermal, electrical, mechanical and dielectric properties of high temperature vulcanized silicone rubber for composite insulators. J. Alloys Compd..

[2] Nazir M.T., Phung B.T., Yu S., Zhang Y., Li S. (2018). Tracking, erosion and thermal distribution of micro-AlN + nano-SiO_2_ co-filled silicone rubber for high-voltage outdoor insulation. High Volt..

[3] Gao Y., Liang X., Bao W., Li S., Wu C., Liu Y., Cai Y. (2019). Effects of liquids immersion and drying on the surface properties of HTV silicone rubber: Characterisation by contact angle and surface physical morphology. High Volt..

[4] Qin G., Shen Z.S., Yu Y.Q., Fan L.D., Cao H.W., Yin C.W. (2019). Effect of Silicone Rubber of a Waste Composite Insulator on Cement Mortar Properties. Materials.

[5] Plesa I., Notingher P.V., Schloegl S., Sumereder C., Muhr M. (2016). Properties of polymer composites used in high-voltage applications. Polymers.

[6] Momen G., Farzaneh M. (2011). Survey of micro/nano filler use to improve silicone rubber for outdoor insulators. Rev. Adv. Mater. Sci..

[7] Zhang Y.J., Zeng X.R., Lai X.J., Li H.Q., Huang X.Y. (2018). Significant improvement of urethane-containing silane on the tracking and erosion resistance of silicone rubber/silica nanocomposite by enhancing the interfacial effect. Polym. Test..

[8] Wu T.Y., Lai X.J., Liu F.J., Li H.Q., Zeng X.R. (2018). Efficiently enhancing the tracking and erosion resistance of silicone rubber by the synergism of fluorine-containing polyphenylsilsesquioxane and ureido-containing MQ silicone resin. Appl. Surf. Sci..

[9] Azizi S., Momen G., Ouellet-Plamondon C., David E. (2020). Performance improvement of EPDM and EPDM/Silicone rubber composites using modified fumed silica, titanium dioxide and graphene additives. Polym. Test..

[10] Shang N.Q., Chen Q.G., Wei X.Z. (2018). Preparation and Dielectric Properties of SiC/LSR Nanocomposites for Insulation of High Voltage Direct Current Cable Accessories. Materials.

[11] Zhang Y.F., Li W., Huang J.H., Cao M., Du G.P. (2020). Expanded Graphite/Paraffin/Silicone Rubber as High Temperature Form-stabilized Phase Change Materials for Thermal Energy Storage and Thermal Interface Materials. Materials.

[12] Chang R.J., Mazeika L. (2000). Analysis of electrical activity associated with inclined-plane tracking and erosion of insulating materials. IEEE Trans. Dielectr. Electr. Insul..

[13] Meyer L.H., Cherney E.A., Jayaram S.H. (2004). The role of inorganic fillers in silicone rubber for outdoor insulation alumina tri-hydrate or silica. IEEE. Electr. Insul. Mag..

[14] Kim S.H., Cherney E.A., Hackam R. (1992). Effects of filler level in RTV silicone rubber coatings used in HV insulators. IEEE Trans. Dielectr. Electr. Insul..

[15] Xue Y., Li X.F., Wang H.S., Zhang D.H., Chen Y.F. (2019). Thermal conductivity improvement in electrically insulating silicone rubber composites by the construction of hybrid three-dimensional filler networks with boron nitride and carbon nanotubes. J. Appl. Polym. Sci..

[16] Liao Y.F., Weng Y.X., Wang J.Q., Zhou H.F., Lin J., He S.J. (2019). Silicone Rubber Composites with High Breakdown Strength and Low Dielectric Loss Based on Polydopamine Coated Mica. Polymers.

[17] He S.J., Hu J.B., Zhang C., Wang J.Q., Chen L., Bian X.M., Lin J., Du X.Z. (2018). Performance improvement in nano-alumina filled silicone rubber composites by using vinyl tri-methoxysilane. Polym. Test..

[18] Xue Y., Li X.F., Wang H.S., Zhao F., Zhang D.H., Chen Y.F. (2019). Improvement in thermal conductivity of through-plane aligned boron nitride/silicone rubber composites. Mater. Des..

[19] Rashid A., Saleem J., Amin M., Ali S.M. (2019). Long-term aging characteristics of co-filled nano-silica and micro-ATH in HTV silicone rubber composite insulators. Polym. Polym. Compos..

[20] Du B.X., Xu H. (2014). Effects of Thermal Conductivity on dc Resistance to Erosion of Silicone Rubber/BN Nanocomposites. IEEE Trans. Dielectr. Electr. Insul..

[21] Chiu H.T., Sukachonmakul T., Kuo M.T., Wang Y.H., Wattanakul K. (2014). Surface modification of aluminum nitride by polysilazane and its polymer-derived amorphous silicon oxycarbide ceramic for the enhancement of thermal conductivity in silicone rubber composite. Appl. Surf. Sci..

[22] Namitha L.K., Ananthakumar S., Sebastian M.T. (2015). Aluminum nitride filled flexible silicone rubber composites for microwave substrate applications. J. Mater. Sci. Mater. Electr..

[23] Zhu B.L., Wang J., Zheng H., Ma J., Wu J., Wu R. (2015). Investigation of thermal conductivity and dielectric properties of LDPE-matrix composites filled with hybrid filler of hollow glass microspheres and nitride particles. Compos. Part B Eng..

[24] Yuan W.H., Xiao Q.Q., Li L., Xu T. (2016). Thermal conductivity of epoxy adhesive enhanced by hybrid graphene oxide/AlN particles. Appl. Therm. Eng..

[25] Ou Z.Z., Gao F., Zhao H.J., Dang S.M., Zhu L.J. (2019). Research on the thermal conductivity and dielectric properties of AlN and BN co-filled addition-cure liquid silicone rubber composites. RSC Adv..

[26] He S.J., He T.F., Wang J.Q., Wu X.H., Xue Y., Zhang L.Q., Lin J. (2019). A novel method to prepare acrylonitrile-butadiene rubber/clay nanocomposites by compounding with clay gel. Compos. Part B Eng..

[27] Xue Y., Li X.F., Zhang D.H., Wang H.S., Chen Y., Chen Y.F. (2018). Comparison of ATH and SiO_2_ fillers filled silicone rubber composites for HTV insulators. Compos. Sci. Technol..

[28] Hougham G., Tesoro G., Viehbeck A. (1996). Influence of Free Volume Change on the Relative Permittivity and Refractive Index in Fluoropolyimides. Macromolecules..

[29] He S.J., Wang J.Q., Hu J.B., Zhou H.F., Nguyen H., Luo C.M., Lin J. (2019). Silicone rubber composites incorporating graphitic carbon nitride and modified by vinyl tri-methoxysilane. Polym. Test..

[30] Yang D., Tian M., Dong Y., Liu H., Yu Y., Zhang L. (2012). Disclosed dielectric and electromechanical properties of hydrogenated nitrile-butadiene dielectric elastomer. Smart Mater. Struct..

[31] Toberer E.S., Baranowski L.L., Dames C. (2012). Advances in Thermal Conductivity. Annu. Rev. Mater. Res..

